# Effect of controlled hypotensive hemorrhage on plasma sodium levels in anesthetized pigs: An exploratory study

**DOI:** 10.14814/phy2.15886

**Published:** 2023-11-27

**Authors:** Rafael T. Krmar, Stephanie Franzén, Leif Karlsson, Helin Strandberg, Susanna Törnroth‐Horsefield, Jesper K. Andresen, Boye L. Jensen, Mattias Carlström, Robert Frithiof

**Affiliations:** ^1^ Department of Physiology and Pharmacology Karolinska Institutet Stockholm Sweden; ^2^ Department of Surgical Sciences, anesthesiology and Intensive Care Uppsala University Uppsala Sweden; ^3^ Department of Women's and Children's Health Karolinska Institutet, Pediatric Endocrinology Unit, Karolinska University Hospital Stockholm Sweden; ^4^ Department of Biochemistry and Structural Biology Lund University Lund Sweden; ^5^ Department of Cardiovascular and Renal Research Institute of Molecular Medicine, University of Southern Denmark Odense Denmark; ^6^ Department of Urology Odense University Hospital Odense Denmark

**Keywords:** aquaporin 2, hemorrhage, hyponatremia, pig model, renin‐angiotensin aldosterone, vasopressin‐neurophysin 2‐copeptin

## Abstract

Perioperative hyponatremia, due to non‐osmotic release of the antidiuretic hormone arginine vasopressin, is a serious electrolyte disorder observed in connection with many types of surgery. Since blood loss during surgery contributes to the pathogenesis of hyponatremia, we explored the effect of bleeding on plasma sodium using a controlled hypotensive hemorrhage pig model. After 30‐min baseline period, hemorrhage was induced by aspiration of blood during 30 min at mean arterial pressure <50 mmHg. Thereafter, the animals were resuscitated with retransfused blood and a near‐isotonic balanced crystalloid solution and monitored for 180 min. Electrolyte and water balances, cardiovascular response, renal hemodynamics, and markers of volume regulation and osmoregulation were investigated. All pigs (*n* = 10) developed hyponatremia. All animals retained hypotonic fluid, and none could excrete net‐free water. Urinary excretion of aquaporin 2, a surrogate marker of collecting duct responsiveness to antidiuretic hormone, was significantly reduced at the end of the study, whereas lysine vasopressin, i.e., the pig antidiuretic hormone remained high. In this animal model, hyponatremia developed due to net positive fluid balance and generation of electrolyte‐free water by the kidneys. A decreased urinary aquaporin 2 excretion may indicate an escape from antidiuresis.

## 1 INTRODUCTION

Acute hypotonic hyponatremia is the most common electrolyte disorder encountered in hospitalized pediatric and adult patients receiving intravenous fluid therapy and is associated with increased levels of the antidiuretic hormone arginine vasopressin (Adrogué et al., 2022; Hoorn et al., 2004; McNab et al., 2015). Furthermore, hyponatremia is associated with adverse outcomes, including an increased risk of death (Adrogué et al., 2022). While in adults, the maintenance fluid's ideal sodium content remains a matter of debate (Van Regenmortel et al., 2021), current pediatric guidelines advise avoiding the use of hypotonic fluid therapy in postoperative and medical acute care settings (Feld et al., 2018).

In surgical patients, a primary goal of intraoperative fluid therapy is to maintain a state of normal body fluid volume, particularly the extracellular fluid volume contained within the vascular system (Sümpelmann et al., 2017). This is a relevant aspect of fluid therapy, as patients with undiagnosed reduction in intravascular volume and increased plasma levels of the antidiuretic hormone arginine vasopressin are at increased risk for developing hyponatremia if hypotonic fluids are given (Holliday et al., 2007). Following this line of reasoning, we have recently conducted a prospective study in children following acute surgery, in whom the amount of blood lost during the surgical procedures was negligible (Roberts et al., 2023). We observed that after correcting participants' extracellular fluid deficit before surgery with a near‐isotonic balanced crystalloid solution (Ringer's acetate solution), the subsequent use of half‐isotonic crystalloid solution as maintenance fluid therapy was not associated with postoperative hyponatremia. In this regard, a previous study conducted in patients that underwent major surgery suggests that the quantity of blood lost during surgery contributes to the pathogenesis of postoperative hyponatremia (Callewart et al., 1994). Understanding the pathogenesis of this electrolyte disturbance might facilitate the development of strategies that can reduce the occurrence of perioperative hyponatremia, which complicates the recovery from many types of surgical procedures (Chung et al., 1986). However, our knowledge of the underlying pathophysiology of hyponatremia following intraoperative blood loss remains incomplete. One aspect hindering this is the lack of suitable in vivo models. Of all the animal models used for renal translational research, pigs offer an advantage since their hemodynamic and renal parameters resemble human physiology and pathophysiology (Franzén et al., 2021; Franzén & Frithiof, 2020).

Against this background, we assumed that blood loss would impact on plasma sodium (pNa) levels in animals exposed to bleeding. Therefore, in this exploratory study, we aimed to investigate the effect of blood loss and treatment of hypovolemia on sodium and water homeostasis in anesthetized pigs by using a controlled hypotensive hemorrhage porcine model. To this end, we examined the factors that may have affected pNa levels during the experimental protocol by consecutively measuring the cardiovascular response and renal hemodynamics as well as key players in volume regulation and osmoregulation, the two adaptative physiological mechanisms that are involved in the development of abnormal pNa levels.

## 2 MATERIALS AND METHODS

### 2.1 Ethical approval and animals

This study was approved by the Local Ethical Committee of the Swedish Board of Agriculture (5.8.18‐02325/2019) and conducted in accordance with the ARRIVE guidelines for animal research (Percie du Sert et al., 2020). The experimental group consisted of 10 female (*n* = 6) and male (*n* = 4) Norwegian Landrace breed/Hampshire/Yorkshire pigs, aged 3–4 months and weighing 22 to 28 kg. They were retrieved from an approved local vendor, fasted overnight at vendor's facility with free access to water, and arrived at the laboratory at 08:00 a.m., two pigs at the time. The experiment was conducted on the same day the animals arrived at our laboratory.

### 2.2 Anesthesia and surgical preparation

All animals were weighed after arrival. The drugs used both for sedation and for anesthesia were the same as described previously (Franzén et al., 2021; Franzén & Frithiof, 2020).

**FIGURE 1:**
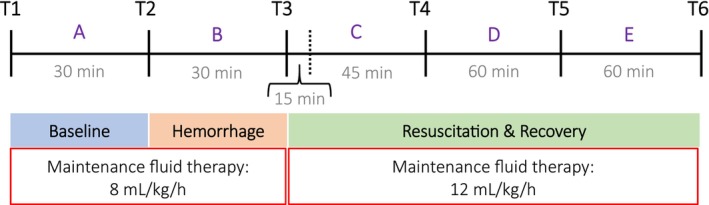
Schematic depicting experimental timeline, within timepoints running from baseline, which started at T1, (i.e., after instrumentation, preparation, and stabilization of the animals), followed by hemorrhage, and resuscitation and recovery, respectively. Euthanasia took place after T6. Within the first 15 min after T3, i.e., during resuscitation, hypovolemia was treated by replacing 60% of the total blood volume withdrawn during hemorrhage with retransfused blood and the remaining 40% with Ringer's acetate solution. Maintenance fluid therapy consisted of a half‐isotonic balanced crystalloid solution (70 mmol/L sodium, 45 mmol/L chloride, 25 mmol/L acetate in 2.5% glucose, Fresenius Kabi®). Blood draws were obtained at each timepoint. Plasma sodium, plasma potassium, plasma chloride, plasma lactate, blood hemoglobin, serum osmolality, cardiovascular parameters, renal blood flow, renal vascular resistance, renal oxygen delivery, and consumption data are presented at T1, T2, T3, and T6, whereas renin, aldosterone, and vasopressin‐neurophysin 2‐copeptin data, at T1, T3, and T6, respectively. Renal function, urine output, and electrolyte‐free water clearance were analyzed at the time intervals A, B, C, D, and E, respectively. Urinary excretion of aquaporin‐2 was analyzed at the time intervals A and E. Urine creatinine, sodium, potassium, and chloride concentration, and urine osmolality were analyzed from an aliquot from each time interval collection.

The animals were sedated with an intramuscular injection of tiletamine‐zolazepam (Zoletil® 6 mg/kg) and xylazine (Rompun® 2.2 mg/kg). After approximately 3 min, the animals were placed in supine position on the surgical table and tracheostomized. They were mechanically ventilated (tidal volume 8–10 mL/kg, respiratory rate 25, PEEP 5 cm H_2_O, and FiO_2_ 40%, respectively) to achieve adequate oxygenation and an arterial pCO_2_ of 4.5–5.5 kPa. A catheter was placed in the ear vein, and a bolus of sedatives was administered intravenously (ketamine 50 mg and morphine 20 mg, respectively). After obtaining controlled breathing, maintenance of general anesthesia was started by a combination of a continuous intravenous heated infusion (38°C) of pentobarbital (8 mg/kg/h) and morphine (0.26 mg/kg/h) dissolved in a half‐isotonic balanced crystalloid solution (70 mmol/L sodium, 45 mmol/L chloride, 25 mmol/L acetate in 2.5% glucose, Fresenius Kabi®) along with the muscle relaxant rocuronium (2.5 mg/kg/h). The animals were continuously monitored for pain reflexes and shivering. If pain reflexes were present, a bolus of ketamine or fentanyl was given. If shivering without pain reflexes was present, a bolus of rocuronium (muscle relaxant) was given. The right carotid artery was catheterized with a single lumen catheter for continuous monitoring of mean arterial pressure (MAP) and blood sampling. The jugular vein on the right side was catheterized with a 3‐lumen catheter for fluid infusion and continuous monitoring of central venous pressure (CVP). In addition, a balloon‐tipped pulmonary artery (PA) catheter (7.5F Swan‐Ganz, Edwards Lifesciences, Irvine, CA) was placed into the right jugular vein and advanced into the PA for monitoring of pulmonary arterial pressure, cardiac output (CO) (mean of 3 measurements), pulmonary capillary wedge pressure (PCWP), and blood sampling. The location of the PA catheter was confirmed by assessing the pressure curve on the monitor derived from the tip of the catheter. After the right atrium was reached, the catheter was carefully advanced into the PA. To confirm correct placement, the balloon was inflated to obtain a PCWP curve on the monitor. The left jugular vein was catheterized with a single lumen catheter for blood sampling. A suprapubic catheter (Foley no. 8. A Datex‐Ohmeda S/5 monitor, Madison, WI) was placed in the bladder for urine collection. The pigs were then turned to lay on their right side. A 10‐cm incision was made from rib to hip to locate and dissect the left kidney. A flow probe (FSB series 4. Transonic, Ithaca, NY) was placed around the renal artery for continuous monitoring of renal blood flow (RBF). A single lumen catheter was then placed into the renal vein for monitoring of renal vein pressure (RVP) and blood sampling. All incisions were closed with sutures (Prolene 3.0). During animals' instrumentation, preparation, and stabilization, i.e., before T1 (Figure 1), all animals received an infusion of 15 mL/kg Ringer's acetate solution to replace any unpredicted surgical blood loss during animals' instrumentation. After surgical preparation, the pigs were allowed to stabilize for 45 min before the experiment commenced. The median time (interquartile range) elapsed from animals' sedation until the start of the experimental protocol (Figure 1) was 116 (110–139) min.

### 2.3 Experimental protocol

The study protocol consisted of three consecutive time intervals as follows: baseline, defined as the time interval between timepoint (T) 1 and T2; controlled hemorrhage, defined as the time interval between T2 and T3; and resuscitation/recovery, defined as the time interval between T3 and T6, respectively, (Figure 1). To facilitate the interpretation of renal function (renal plasma creatinine clearance), urine output, and electrolyte‐free water clearance (ECH₂O) data, these variables were analyzed in five consecutive time intervals as follows: interval A, corresponding to baseline, interval B, corresponding to controlled hemorrhage, and then the intervals C, D, and E, corresponding to resuscitation and recovery, respectively, (Figure 1).

During baseline, all animals received a continuous infusion of a maintenance fluid and electrolyte therapy consisting of a half‐isotonic balance crystalloid solution (70 mmol/L sodium, 45 mmol/L chloride, 25 mmol/L acetate in 2.5% glucose, Fresenius Kabi®) at a dose of 8 mL/kg/h. Maintenance fluid therapy was aimed at replacing animals' insensible and sensible water and electrolyte losses during the conduct of the study. During hemorrhage, blood was continuously removed at 2 mL/kg/min to achieve a MAP of <50 mmHg within 10–15 min. Blood was then withdrawn slowly to maintain a MAP of <50 mmHg for a total time of 30 min. Before achieving a MAP of <50 mmHg, the hemorrhaged blood was stored in a heparinized blood collection bag for subsequent use in resuscitation. Within the first 15 min after hemorrhage period, the total blood volume lost during hemorrhage was replaced (resuscitation). Sixty percent of this volume was composed of animal's blood and 40% of Ringer's acetate solution (131 mmol/L sodium, 4 mmol/L potassium, 1 mmol/L magnesium, 112 mmol/L chloride, 2 mmol/L calcium, 30 mmol/L acetate; Fresenius Kabi®), respectively. The infusion of maintenance fluid therapy during resuscitation and recovery period, i.e., from T3 to T6, was increased to a dose of 12 mL/kg/h. After the experimental protocol was finished, pigs were euthanized with potassium chloride.

### 2.4 Electrolyte and water balance

From the start of hemorrhage (T2) until the end of study (T6), electrolytes (sodium, potassium, and chloride) and fluid intakes were calculated from all given intravenous fluid, including maintenance fluid therapy, fluid administered during resuscitation (i.e., blood and Ringer's acetate solution), 0.9% NaCl solution (154 mmol/L sodium, 154 mmol/L chloride) given to measure CO, and anesthetic medications. Electrolytes and fluid output were determined from samples from all collected urine, which was collected continuously by means of a suprapubic catheter. The volume of blood that was not retransfused, i.e., 40% of blood withdrawn during hemorrhage, was also considered in the electrolyte and water balance. Plasma sodium, potassium, and chloride values measured at T2 were used in the blood component of electrolyte balance. Fluid lost from the respiratory system and skin were not included. The electrolyte and water balances were calculated by subtracting total fluid output from total fluid input.

### 2.5 Sample collection and laboratory analysis

At every timepoint (Figure 1), i.e., from the start of baseline period (T1) to the end of the study (T6), blood gas (used for the direct measurement of pNa, plasma potassium, plasma chloride, and plasma lactate, respectively), plasma creatinine, blood hemoglobin, and serum osmolality were analyzed. The urine that was collected at the end of interval A, B, C, D, and E (Figure 1) was recorded and measured volumetrically, and urine creatinine, sodium, potassium, and chloride concentration and urine osmolality were analyzed from an aliquot from each collection. The serum and the urine osmolality were measured by a freezing point depression technique. Determination of routine blood and urine analyses was performed according to accredited hospital clinical laboratory procedures at the Clinical Chemistry Laboratory, Uppsala University Hospital, Uppsala, Sweden. Arterial, central venous, and renal venous blood gasses collected during the experimental protocol were processed on Radiometer ABL 700 Copenhagen, Denmark.

### 2.6 Plasma‐renin, aldosterone, and vasopressin‐neurophysin 2‐copeptin analysis

As previously described (Lindestam et al., 2019), the plasma‐renin concentration was determined from EDTA plasma that was incubated with plasma from a nephrectomized sheep followed by radioimmunoassay of angiotensin I generated through the antibody trapping method as described by Poulsen and Jorgensen (1974). The validity of using sheep plasma for renin concentration measurement with pig plasma renin was validated in two ways: A control pig EDTA‐plasma sample was incubated for 3 h at 37°C with and without addition of substrate. In the presence of substrate, the same sample in the same incubation time (3 h) generated 1,34 ng/tube ANGI, whereas in the absence of substrate, this generated 0,152 ng/tube ANGI. Accuracy was tested by a dilution series of plasma in consecutive steps of 1:2. Until 1:8 dilution, the series was linear. Concentrations were measured by the rate of angiotensin I formation and standardized in terms of international units per liter (IU/L) based on the World Health Organization International Standard (ref. no. 68–356; National Institute for Biological Standards and Control, Hertfordshire, UK), with samples of 0.05 IU/L included in every run of the plasma‐renin concentration assay. The inter‐assay coefficients of variation of two different human plasma pool samples across the six independent assay runs were 13.6% and 13.8%, respectively.

Plasma aldosterone was determined by ELISA (MS E‐5200, LDN, Labor Diagnostika Nord, Germany). Human EDTA plasma pool was used as an internal inter‐assay standard. The inter‐assay coefficient of variation across the five independent assays in the present study was 6.5%.

Vasopressin‐neurophysin 2‐copeptin, the precursor of lysine vasopressin, i.e., the pig antidiuretic hormone (Sawyer et al., 1960), was determined according to the protocol from the manufacturer (Abbexa catalog No: abx512712). We determined the in‐house measuring range by serial dilution of two samples from two pigs 1:5, 1:10, 1:50, and 1:250, respectively. After this initial run, all samples were pre‐diluted 1:20 (10 μL sample in 190 μL diluent buffer) which resulted in values well within the standard curve in a microplate (#650201, Greiner bio‐one). From the dilution, 50 μL was aliquoted into 50 μL diluent buffer on the plate provided from the kit. Incubation time was 2 h at 37°C. Plates were read at 450 nm. The intra‐assay variation was tested in‐house with two samples from two pigs with low and medium levels of vasopressin‐neurophysin 2‐copeptin in a 1:50 dilution (*n* = 8, repetitions of each). Coefficient of variation was 6,2% with low‐range sample and 6,4% with medium‐range sample. Furthermore, linearity/accuracy of the assay was tested with four samples. They were diluted 2‐fold in the range (1:5, 1:10, 1:20, 1:40, 1:80, 1:160, 1:320, and 1:640, respectively), and four measurements from each sample within the range of the standard curve were used for calculation. Linearity: CV = 14,8%.

### 2.7 Urinary excretion of aquaporin‐2 (AQP2)

The amount of AQP2 in the urine was quantified using a sandwich ELISA assay. For the assay, urine samples were pre‐treated by centrifugation at 1000 × *g* for 10 min to remove cellular debris, and 900 μL of each urine sample was concentrated in a Vivaspin® 500 concentrator with 10 kDa cutoff (Sartorius). PBS was added to a final volume of 300 μL and 150 μL of 0,03% SDS in PBS‐T was added (0,01% final SDS concentration) after which the samples were left to incubate at room temperature for 10 min. A 1:2 dilution series (100‐0,046 nM) of recombinantly expressed and purified human full‐length AQP2 (Roche et al., 2017) in sample diluent buffer (PBS‐T, 0.2% BSA) was used as standards, and the spinach aquaporin SoPIP2;1 (Törnroth‐Horsefield et al., 2006) was used as negative control (25 nM in sample diluent buffer).

A 96‐well polystyrene plate (ThermoFisher) was coated with a mouse monoclonal antibody directed against the human AQP2 C‐terminus (Invitrogen, MA5‐31630) diluted 1:5000 in carbonate buffer pH 9.4 by incubating for 2 h at room temperature with gentle shaking. After 3 × 5 min of washing with PBS‐T, the plate was blocked with 1% BSA in PBS‐T for 1 h at 37°C. 100 μL of urine samples and standards were added to the plate in triplicate, and the plate was left to incubate overnight at 4°C. Following washing as above, a rabbit polyclonal antibody directed against the human AQP2 N‐terminus (Origene, AP08304PU‐N) diluted 1:500 in sample diluent buffer was added, and the plate was incubated for 2 h with gentle shaking. The plate was washed as above and incubated with a goat anti‐rabbit IgG HRP‐conjugated antibody (Invitrogen, 31460) diluted 1:5000 in sample diluent buffer for 1 h at room temperature with gentle shaking. After 6 × 5 min of washing with PBS‐T, 100 μL TMB (3,3′,5,5′‐Tetramethylbenzidine, Sigma Aldrich) was added to each well, and the plate was incubated for 30 min at room temperature with gentle shaking. The reaction was stopped by adding 100 μL of 0.18 M H_2_SO_4_, and the absorbance at 450 nm was determined in a CLARIOStar plate reader (BMG Labtech) using sample diluent buffer as blank.

The absorbance values for the AQP2 standards were used to fit a standard curve in GraphPad Prism (version 9.4.1) (R^2^ = 0.9972) from which the AQP2 concentration in the urine samples was calculated. For samples where the AQP2 concentration was estimated to be below the range of the standard curve, the concentration of the lowest standard was used to indicate the maximum AQP2 concentration in these samples rather than the absolute value.

### 2.8 Renal hemodynamics, function, and oxygenation

Parameters that were registered and calculated for results included RBF, renal vascular resistance (RVR), measured renal plasma creatinine clearance (CrCl), urine output, ECH₂O, renal oxygen delivery (RDO_2_), and renal oxygen consumption (RVO_2_), respectively.

### 2.9 Cardiovascular variables

Parameters that were registered and calculated included heart rate, MAP, CVP, PCWP, and CO, respectively.

### 2.10 Calculations

The electrolyte‐free water (mL) received from T2 until the end of study (T6), which represents the volume of pure water contained in the fluids given relative to pNa levels at T2, was calculated as follows:

$$\begin{array}{c} \left[\text{Total volume infused}\;\left(L\right)-\left(\text{total sodium infused in}\right.\right.\\ \left.\;\text{mmol}/\mathrm{pNa}\;\mathrm{at}\left.\;\text{T2}\right)\right]\times 1000 \end{array}$$

The rate of urine output (mL/min) was calculated by dividing the volume of urine produced by the number of minutes that have elapsed since the bag was last emptied.

Effective osmolal clearance (ECOsm), which refers to the clearance of only effective osmoles, i.e., sodium, potassium, and their accompanying anions, was calculated as follows (Shimizu et al., 2002):

$$\text{ECOsm}\;\left(\mathrm{mL}/\min\right)=u\;\left(\mathrm{Na}+K\right)\times V/\mathrm{pNa}$$

where u (Na + K) represents urinary sodium plus potassium concentration (mmol/L) from urine that was carefully collected at interval A, B, C, D, and E, and which were recorded and measured volumetrically; V, rate of urine output (mL/min); pNa, plasma sodium concentration at the end of each interval, i.e., at T2, T3, T4, T5, and T6, respectively.

ECH₂O, a method used to determine whether net‐free water is reabsorbed from or added to the tubular fluid during urine concentration, and thereby its effect on pNa was calculated as follows:

$$\mathrm{ECH}₂O\;\left(\mathrm{mL}/\min\right)=V-\text{ECOsm}$$

CrCl (mL/min) was calculated as follows:

$$\left(\mathrm{uCr}\times V\right)/\mathrm{pCr}$$

where uCr and pCr represent urine and plasma creatinine, respectively.

RVR (mmHg/mL/min) was calculated as follows:

$$\left(\mathrm{MAP}-\mathrm{RVP}\right)/\mathrm{RBF}$$

The O_2_ content in blood was calculated as follows:

$$\left({\mathrm{SO}}_{2}\times \text{hemoglobin}\times 1.39\right)+\left({\mathrm{pO}}_{2}\times 0.003\right)$$

where SO_2_ represents oxygen saturation and pO_2_ represents partial pressure of oxygen, respectively.

RDO_2_ (mL/min) was calculated as follows:

$$\left(\mathrm{RBF}\times \text{arterial}\;O_{2}\;\text{content}\right)/1000$$

and RVO_2_ (mL/min) as follows:

$$\begin{array}{c} \left(\left[\text{arterial}\;O_{2}\;\text{content}-\text{renal venous}\;O_{2}\;\text{content}\right]\times \mathrm{RBF}\right)/1000 \end{array}$$

### 2.11 Statistical analysis

Statistical analyses were performed using GraphPad Prism 9 (version 9.5.1). All variables are presented as mean (±SD) or as median (interquartile ranges). Paired *t*‐test was carried out to compare individual differences between baseline and at the end of the experimental protocol. Repeated measures one‐way analysis of variance (ANOVA), followed by Tukey's multiple comparisons test to compare variables between different timepoints, was used. Two‐tailed *p* values < 0.05 after correction for multiple comparisons were considered significant.

## 3 RESULTS

A schematic overview of the experimental study protocol is shown in Figure 1. All animals (*n* = 10) survived the experimental protocol and were included in the statistical analysis.

### 3.1 Electrolyte and water balance

**FIGURE 2:**
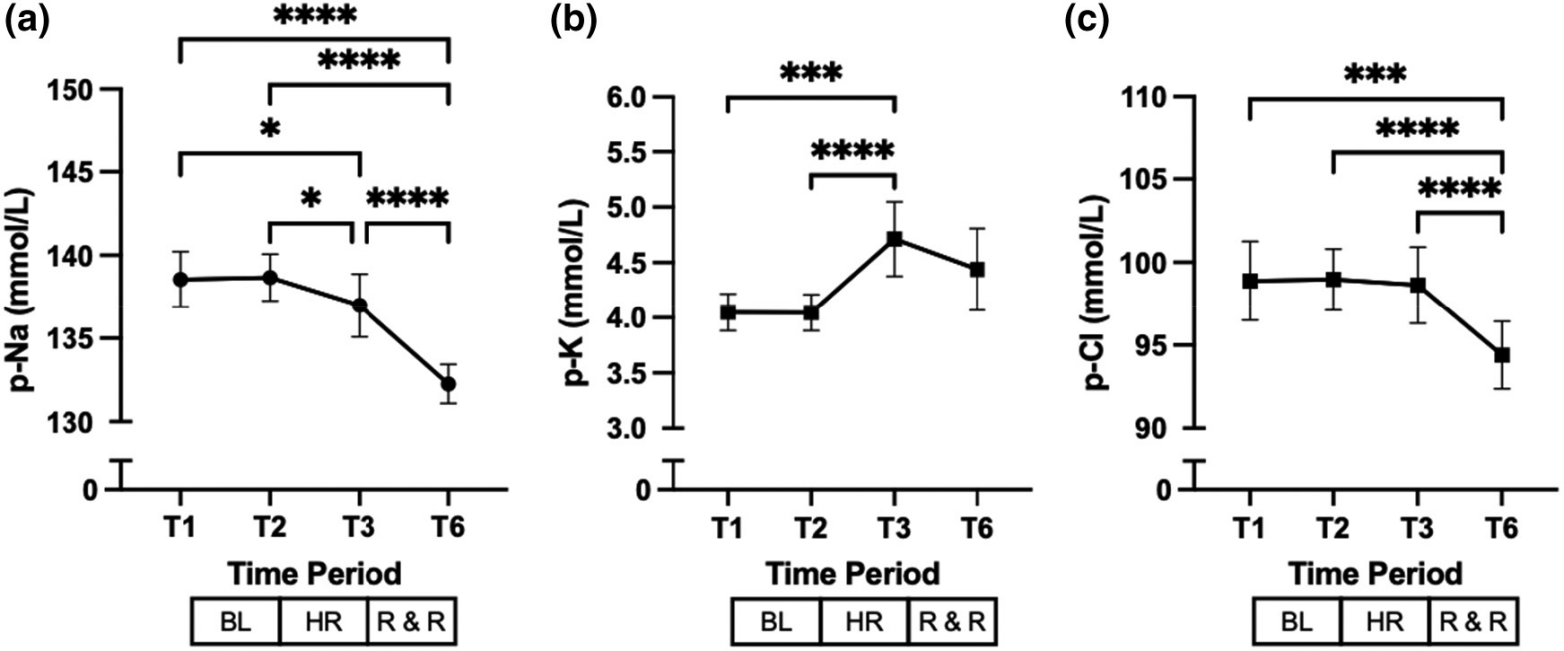
Effect of hemorrhage, resuscitation, and recovery on electrolytes. (a) p‐Na, plasma sodium; (b) p‐K, plasma potassium; (c) p‐Cl, plasma chloride. T denotes timepoints, BL baseline, HR hemorrhage, and R & R resuscitation and recovery, respectively. Values are mean (±SD). *p < 0.05, ***p ≤ 0.001, and ****p ≤ 0.0001.

The median (interquartile range) total fluid received from the start of hemorrhage to the end of the study, which includes the resuscitation and recovery period (Figure 1), was 2127 (2056–2210) mL, and the median urine volume was 601 (547–654) mL, respectively, (Table 1). During this period, the balance for water, sodium, and chloride was positive, whereas it was negative for potassium (Table 1). The calculated median electrolyte‐free water contained in the fluids that were administered from the start of hemorrhage until the end of the study was 643 (619–671) mL, which corresponds to a median‐free water infusion rate of 0.13 (0.13–0.13) mL/kg/min. As can be seen from Table 1, the amount of water and sodium retained was hypotonic, indicating that there was an increase in the retained amount of infused electrolyte‐free water relative to the pNa concentration measured before the start of hemorrhage (Figure 2a).

**TABLE 1:** Data on water and electrolyte balance (*n* = 10).

From the start of hemorrhage to the end of study	Water (mL)	Sodium (mmol)	Potassium (mmol)	Chloride (mmol)
Infused	2127 (2056–2210)	208 (201–216)	2 (2–2)	167 (161–173)
Excreted	601 (547–654)	75 (71–79)	24 (20–27)	55 (43–65)
Balance	+1477 (1461–1553)	+133 (125–138)	−23 (−24 to −18)	+114 (106–120)

*Note*: Results are presented as medians (IQRs). Decimals were rounded to the nearest integer.

### 3.2 Effect of hemorrhage, resuscitation, and recovery on plasma electrolytes

The course of pNa and plasma chloride after controlled hypotensive hemorrhage showed a steady and significant decrease until the end of study (Figure 2a,c, respectively). At T1, i.e., approximately 2 h after animals' instrumentation, preparation, and stabilization, all animals had pNa levels within normal limits (≥135 and ≤145 mmol/L). At the end of the study, hyponatremia (defined as pNa < 135 mmol/L) was observed in all animals (median 132, range 131–133 mmol/L, Figure 2a), which was mild, i.e., pNa ≥130 <135 mmol/L in all the pigs (Spasovski et al., 2014). Despite a negative balance (Table 1), plasma potassium increased significantly after hemorrhage, which was followed by no significant change after resuscitation (Figure 2b).

### 3.3 Effect of hemorrhage, resuscitation, and recovery on hormones that regulate volume and on markers of volume

**FIGURE 3:**
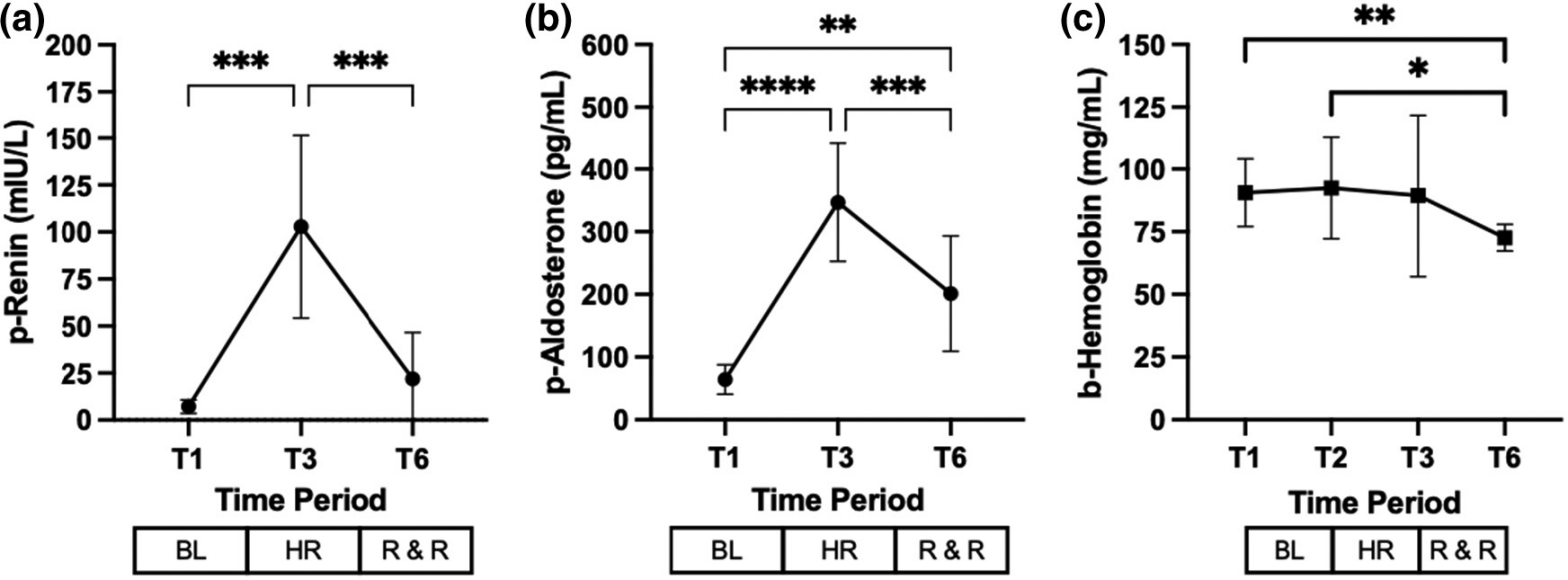
Effect of hemorrhage, resuscitation, and recovery on markers of volume regulation. T denotes timepoints, BL baseline, HR hemorrhage, and R & R resuscitation and recovery, respectively. Values are mean (±SD). *p < 0.05, **p ≤ 0.01, ***p ≤ 0.001, and ****p ≤ 0.0001.

As compared to baseline values, both renin and aldosterone levels increased significantly after controlled hemorrhage, which was followed by a significant decrease at the end of the study. At this timepoint, renin levels were like baseline values, whereas aldosterone levels remained significantly higher as compared to baseline values (Figure 3a,b).

After hemorrhage and during resuscitation and recovery period, blood hemoglobin levels showed a significant decrease (Figure 3c).

### 3.4 Effect of hemorrhage, resuscitation, and recovery on markers of osmoregulation

**FIGURE 4:**
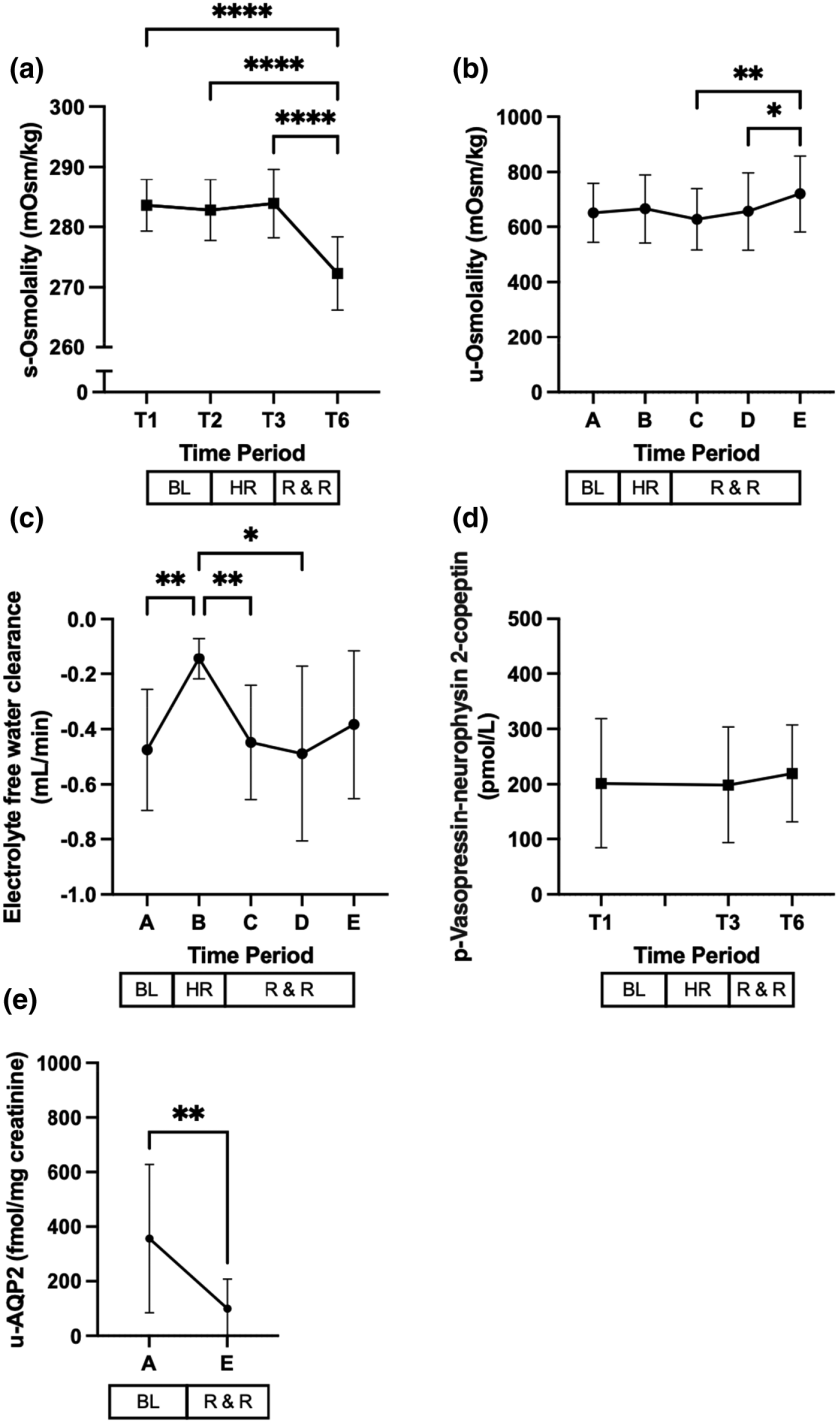
Effect of hemorrhage, resuscitation, and recovery on markers of osmoregulation. T denotes timepoints. Time interval A corresponds to baseline (BL), interval B corresponds to hemorrhage (HR), and intervals C, D, and E, correspond to resuscitation and recovery (R & R), respectively. Values are mean (±SD). *p < 0.05, **p ≤ 0.01, and ****p ≤ 0.0001.

There was a decrease in serum osmolality, which became significant at the end of the study (Figure 4a). This was accompanied by a significant increase in urine osmolality (Figure 4b).

At baseline, the median ECH₂O was negative in all animals and remained so until the end of the study (Figure 4c). Of note, during hemorrhage, ECH₂O increased, though this was a transient event, since during resuscitation and recovery levels returned to baseline values (Figure 4c).

As compared to baseline values, pig vasopressin‐neurophysin 2‐copeptin levels, the precursor of lysine vasopressin, i.e., the pig antidiuretic hormone (Sawyer et al., 1960), did not significantly change after hemorrhage (Figure 4d).

Data for aquaporin 2 (AQP2) levels were available in nine animals, where there was a significant decrease in the urinary excretion of AQP2 at the end of the study with respect to baseline values (Figure 4e).

### 3.5 Effect of hemorrhage, resuscitation, and recovery on hemodynamic and metabolic parameters

**FIGURE 5:**
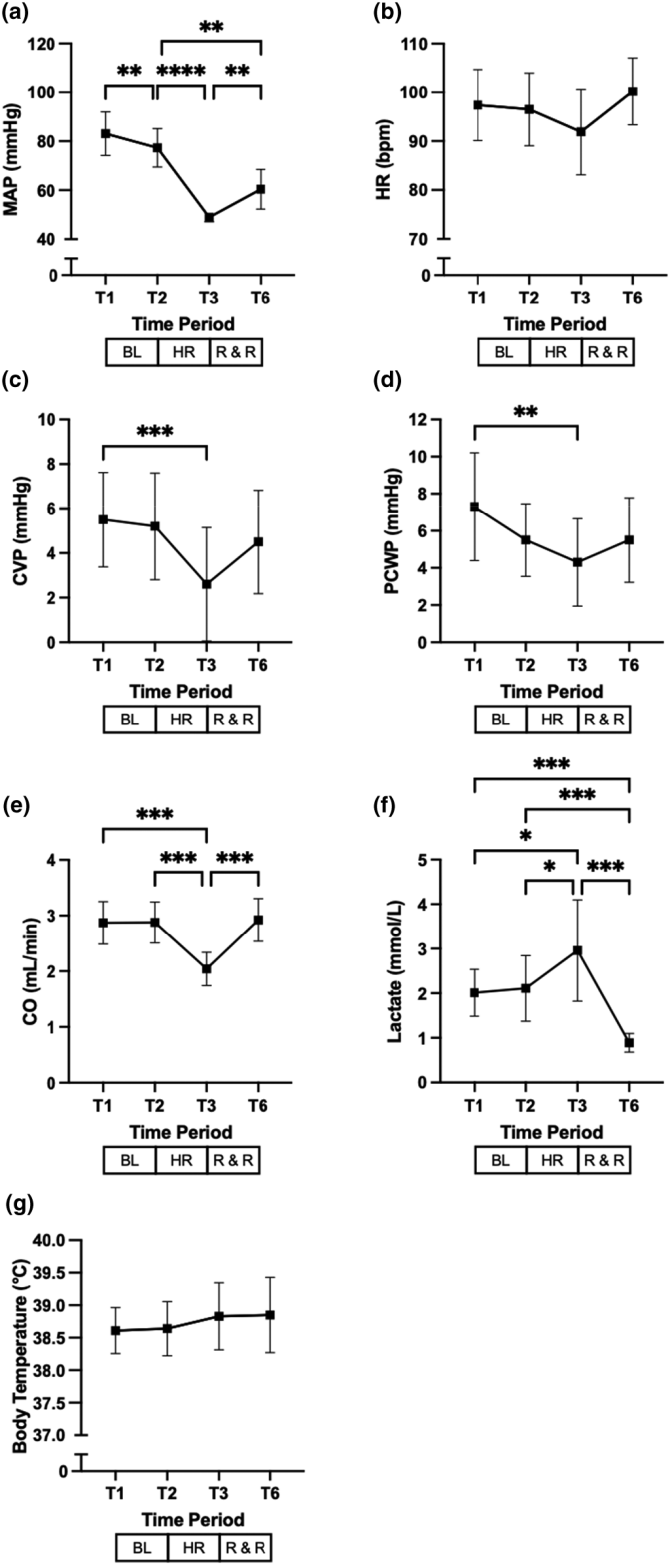
Effect of hemorrhage, resuscitation, and recovery on hemodynamic and metabolic parameters. MAP, mean arterial pressure; HR, heart rate; CVP, central venous pressure; PCWP, pulmonary capillary wedge pressure; CO, cardiac output. T denotes timepoints, BL baseline, HR hemorrhage, and R & R resuscitation and recovery, and bpm beats per minute, respectively. Values are mean (±SD). *p < 0.05, **p ≤ 0.01, ***p ≤ 0.001, and ****p ≤ 0.0001.

Hemorrhage significantly decreased MAP, CVP, PCWP, CO (Figure 5a,c–d, respectively). Heart rate was not significantly affected during the conduct of the study (Figure 5b).

As shown in Figure 5, changes in CVP, which mirrored the observed reductions in MAP caused by hemorrhage, were associated to comparable reductions of CO.

Plasma lactate significantly increased after controlled hemorrhage, whereas it decreased to levels below baseline values after resuscitation (Figure 5f). During the study period, body temperature did not change significantly (Figure 5g).

### 3.6 Effect of hemorrhage, resuscitation, and recovery on renal parameters

**FIGURE 6:**
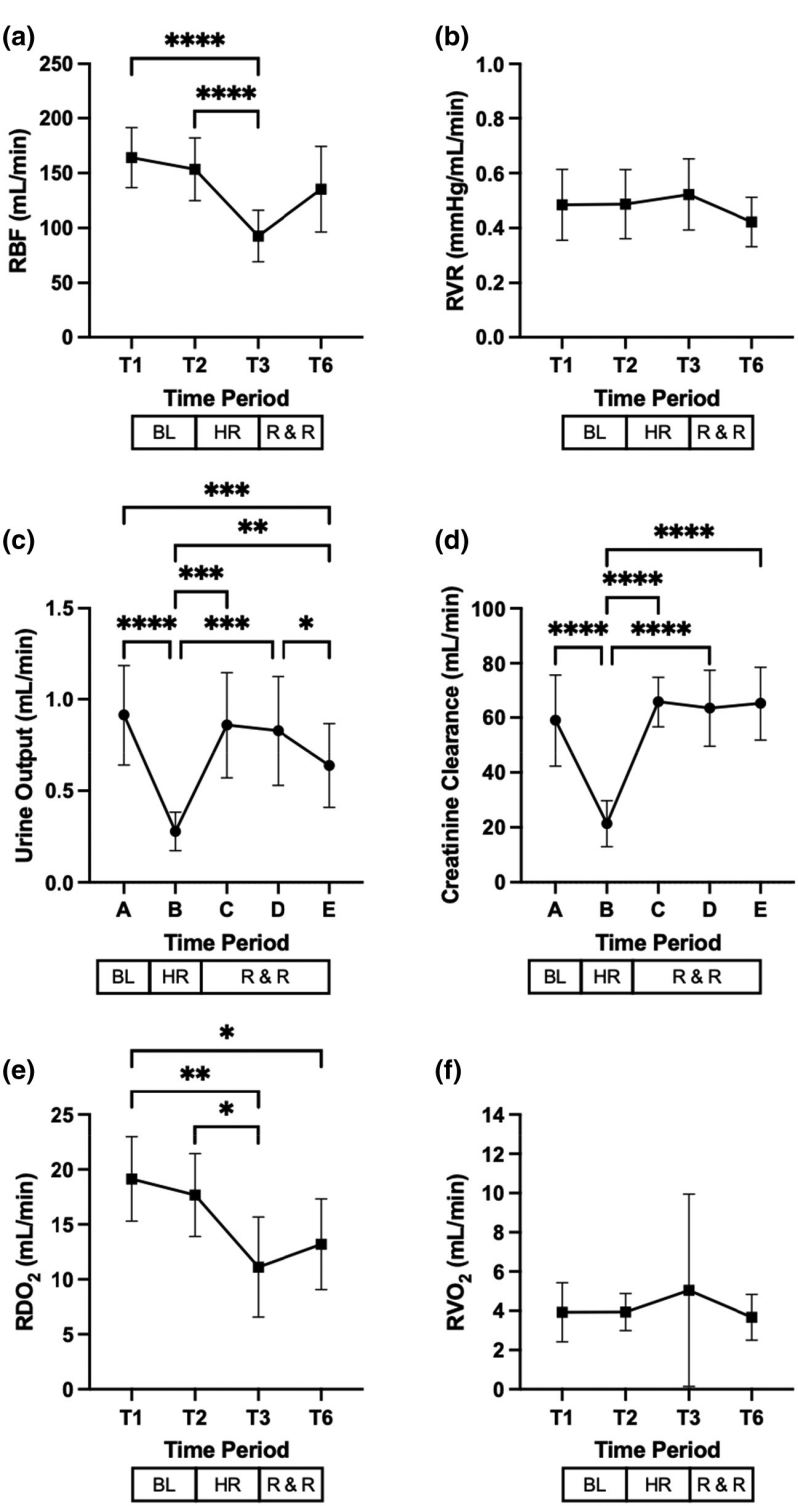
Effect of hemorrhage, resuscitation, and recovery on renal parameters. RBF, renal blood flow; RVR, renal vascular resistance; RDO_2_, renal oxygen delivery; RVO2, renal oxygen consumption. T denotes timepoints. Time interval A corresponds to baseline (BL), interval B corresponds to hemorrhage (HR), and intervals C, D, and E, correspond to resuscitation and recovery (R & R), respectively. Values are mean (±SD). *p < 0.05, **p ≤ 0.01, ***p ≤ 0.001, and ****p ≤ 0.0001.

Hemorrhage caused a significant decrease in RBF, which recovered after resuscitation (Figure 6, panel A). RVR remained unchanged during the study (Figure 6b). Controlled hemorrhage also caused a transient and significant decrease in urine output. This variable recovered after resuscitation, though at the end of the study, it remained below baseline values (Figure 6c). CrCl decreased significantly during controlled hemorrhage and returned to baseline values after resuscitation (Figure 6d).

A significant reduction in RDO_2_ was observed due to hemorrhage, whereas RVO_2_ remained unaffected (Figure 6e,f, respectively).

## 4 DISCUSSION

The sequence of hemorrhage, resuscitation, and recovery and the development of hyponatremia observed in our study suggest that our intervention caused abnormally low pNa levels in previously normonatremic anesthetized and mechanically ventilated pigs (Figure 2a and Figure 4a, respectively).

During the experimental protocol, the calculation of electrolyte‐free water indicated that all animals received a certain amount of pure water. From baseline until the end of the study, the electrolyte‐free water clearance values were negative in all animals (Figure 4c), which in the presence of high plasma vasopressin‐neurophysin 2‐copeptin levels, indicates that the kidneys generated electrolyte‐free water. This is expected to have had a meaningful impact on animals' pNa levels (Berl, 2008). This is also captured in the assessment of the net balance of electrolytes and water, which shows hypotonic gains, i.e., the reabsorbed fluid contained a low solute concentration and high water concentration compared to body fluids (Table 1). These results differ from our previous study conducted in children with acute appendicitis (Roberts et al., 2023). In that study, participants had also received near‐isotonic and half‐isotonic intravenous fluid therapy. However, they were able to excrete net‐free water and the amount of water and sodium retained was isotonic, despite having received electrolyte‐free water. It is worth mentioning that the assumed fluid losses before surgery were slow and represented a state of mild dehydration. Neither were they hemodynamically compromised, while their intraoperative bleeding was negligible (Roberts et al., 2023). Therefore, controlled hypotensive hemorrhage, which not only reduced rapidly animals' blood volume, but also significantly affected their systemic and renal hemodynamics, may have accounted for the observed changes in animals' pNa.

Renin and aldosterone levels increased after hemorrhage (Figure 3a,b, respectively). This is an expected event, since the renin‐angiotensin aldosterone system is activated in response to a decrease in the effective circulating volume in order to correct blood volume and stabilize perfusion to key organs such as the brain, heart, and the kidneys (Cano et al., 1994; Harrison‐Bernard, 2009; Quinn & Williams, 1988).

In our experiments, the replacement of the total blood volume withdrawn during hemorrhage was intended to correct animals' hypovolemia. In fact, resuscitation was followed by a significant recovery in MAP and CO as well as an increase in CVP and PCWP, (Figure 5a,c–e, respectively).

Heart rate was not affected by hemorrhage (Figure 5b). Although anesthesia might have affected heart rate (Frithiof et al., 2006), it is worth noting that cardiac responsiveness in conditions of central hypovolemia may significantly differ between pigs and humans. Our observation is in accordance with a previous study designed to evaluate hemodynamic variable in anesthetized pigs submitted to graded hemorrhage and retransfusion, in which heart rate remained stable until blood was withdrawn to a total of −30 mL/kg (Dalibon et al., 1999). Considering this, the median blood volume that was withdrawn during hemorrhage in our study was 19.5 (15–23) mL/kg, which is lower than in the aforementioned study.

As mentioned above that water retention was a significant contributor to hyponatremia. This is also highlighted by a significant decrease in blood hemoglobin levels observed during the experiment (Figure 3c) (Chan et al., 2009; Nose et al., 1988).

The significant increase in plasma lactate levels observed after hemorrhage may be attributed to a decrease in the effective circulating arterial blood volume due to the combination of acute blood loss and hypotension (Kreimeier, 2000). Plasma lactate levels returned to baseline values by treating hypovolemia (Figure 5f), which may be indicative of efficient energy production, sufficient to maintain euthermia (Figure 5e,f, respectively), indicating that tissue oxygenation was not jeopardized during the conduct of the experimental protocol (Fuller & Dellinger, 2012; Kaplan et al., 2001).

Circulating plasma vasopressin‐neurophysin 2‐copeptin levels, the precursor of lysine vasopressin, were not affected by hemorrhage (Figure 4d), though their levels were already high at baseline. Considering that one of the basic mechanisms involved in the homeostatic control of plasma arginine vasopressin is related to blood volume (Gauer & Henry, 1963), the lack of reactivity was unexpected since plasma arginine vasopressin levels increase significantly after bleeding (Bankir et al., 2017). Indeed, in an experimental baboon model of hemorrhagic shock, copeptin, a stable surrogate of the antidiuretic hormone arginine vasopressin (Szinnai et al., 2007), increased significantly after controlled bleeding (Morgenthaler et al., 2007). It should be noted that the vasopressin‐neurophysin 2‐copeptin levels observed in our study were similar to the copeptin levels observed after bleeding in the baboon model (Morgenthaler et al., 2007). It is worth mentioning that vasopressin‐neurophysin 2‐copeptin levels at baseline were taken approximately 2 h after animals' sedation, i.e., after instrumentation, preparation, and stabilization of the animals (Figure 1). Since the release of arginine vasopressin is a natural response to anesthesia and surgery (Cochrane et al., 1981; Philbin & Coggins, 1980), we reason that this may have ultimately affected vasopressin‐neurophysin 2‐copeptin levels before the start of our experimental protocol. A study conducted in dogs designed to evaluate the effects of anesthesia, surgery, and intravenous administration of fluids on plasma arginine vasopressin levels provides additional support to our speculation (Hauptman et al., 2000).

At the same time, it is useful to note that in our previous studies conducted in surgical patients, we observed that after correcting their extracellular fluid deficit, the degree of water retention decreased despite the high circulating plasma levels of the antidiuretic hormone arginine vasopressin at the end of surgery (Lindestam et al., 2019; Roberts et al., 2023). We reasoned that this observation suggests the appearance of a mechanism aimed at limiting dilutional hyponatremia. We measured urinary excretion of AQP2 as a surrogate marker of collecting duct responsiveness to arginine vasopressin (Elliot et al., 1996). Urinary excretion of AQP2 was reduced at the end of the study (Figure 4e), whereas plasma vasopressin‐neurophysin 2‐copeptin levels remained unchanged (Figure 4d). Thus, the significant reduction in the urinary excretion of AQP2 together with less negative, though not significant, ECH_2_O values at the end of the study (Figure 4c) may suggest the presence of a regulatory mechanism intended to attenuate the reabsorption of free water, i.e., an escape from antidiuretic hormone arginine vasopressin‐induced antidiuresis (Ecelbarger et al., 1997). Although a tempting explanation, additional studies investigating the regulation of AQP2 expression in renal tissue are needed to confirm this line of thought (Lee et al., 2018; Nielsen et al., 2002).

Urine output and renal function, i.e., renal plasma creatinine clearance, were significantly reduced after hemorrhage, which should be regarded as a renal response to a low flow state (Figure 6c,d, respectively). The decrease in these parameters was proportional to the magnitude of the decrease in RBF (Figure 6a). Furthermore, replacement of the total blood volume lost during hemorrhage was linked to recovery of the CO and RBF (Figure 5e and Figure 6a, respectively). Thus, the hemodynamic beneficial effects of resuscitation were reflected in the increase of urine output and renal function, which might be considered as an index of renal performance after fluid replacement following hemorrhage (Sondeen et al., 1990). The RVR did not change significantly during the conduct of the study (Figure 6b). Anesthesia may have suppressed the renal vascular response to hypovolemia observed in our study (Frithiof et al., 2006).

Oxygen consumed by the kidney is primarily coupled to sodium reabsorption through Na^+^, K^+^‐ATPase molecules (Jørgensen, 1980). During hemorrhage, we observed a significant decrease in RDO_2_ in all animals, whereas RVO_2_ remained stable, and the net balance of sodium was positive (Figure 6e,f and Table 1, respectively). The former is an expected event following hemorrhage (Koons et al., 2020). The latter indicates that in our model, despite the observed decrease in oxygen delivery, oxygen demand was however matched.

A limitation of our exploratory study is the omission of a control group that received only a half‐isotonic balanced crystalloid solution. In our previous animal studies, however, we did not observe significant changes in pNa after instrumentation, preparation, and stabilization of the animals (Frithiof et al., 2006; Luther et al., 2023). Therefore, we trust that the lack of a control group that did not undergo controlled hemorrhage does not jeopardize the level of evidence and the conclusions derived from our observations. Nonetheless, the lack of a group treated with isotonic crystalloid fluid possesses a challenge, since we do not know if hyponatremia would have been prevented, had we used isotonic crystalloid solutions for the treatment of both hypovolemia and animals' maintenance needs. Additional hypothesis‐testing studies are needed to evaluate whether this is the case.

## 5 CONCLUSION

In this exploratory study, we observed that hyponatremia developed after controlled hypotensive hemorrhage in anesthetized pigs, which produced important alterations of cardiovascular and renal hemodynamics as well as of volume regulation and osmoregulation homeostatic mechanisms. We further observed that the urinary excretion of AQP2 was significantly reduced at the end of the study, which in the context of high circulating plasma vasopressin‐neurophysin 2‐copeptin levels, may suggest an escape from antidiuresis. Further investigations are needed to evaluate this possibility.

Finally, the interpretation of our results provides insights into the pathogenesis of hemorrhage‐induced hyponatremia. Our observations may contribute to a better understanding of the potential implications of intraoperative bleeding on pNa levels in surgical patients. However, additional studies are needed to confirm whether our observations translate to humans.

## AUTHOR CONTRIBUTIONS

Original idea, R.T.K. and R.F.; experimental design, R.T.K., S.F., M.C., and R.F.; data collection, R.T.K., S.F., and R.F.; laboratory analysis, H.S., S.T‐H., M.C., J.K.A., and B.L.J.; statistical analysis, L.K., R.T.K., and M.C.; writing original draft preparation, R.T.K.; preparation of tables and figures, R.T.K. and M.C.; review and editing, all authors. All authors have critically revised the manuscript for important intellectual and scientific content and approved the final version.

## FUNDING INFORMATION

This work was funded by the Swedish Research Council (2020‐01645 to M.C. and 2014‐02569 and 2014‐07606 to R.F.), the Swedish Heart and Lung Foundation (20210431 to M.C.), NovoNordisk (2019#0055026 to M.C.), and by Research Funding from the Karolinska Institutet, Stockholm, Sweden.

## CONFLICT OF INTEREST STATEMENT

R.T.K. declares that he is employee of Swedish Orphan Biovitrum AB (Sobi™), which had no role in funding, study design, data collection and analysis, decision to publish, or preparation of the manuscript. All other authors declare that there are no relevant financial or nonfinancial competing interests to report.

## ETHICS STATEMENT

Ethical approval was given by the Local Ethical Committee of the Swedish Board of Agriculture (5.8.18‐02325/2019).

## Data Availability

The datasets generated during the current study are available from the corresponding author on reasonable request.
